# Hemodynamic Indexes Derived from Computed Tomography Angiography to Predict Pulmonary Embolism Related Mortality

**DOI:** 10.1155/2014/363756

**Published:** 2014-06-18

**Authors:** Gregor John, Christophe Marti, Pierre-Alexandre Poletti, Arnaud Perrier

**Affiliations:** ^1^Department of Internal Medicine, Rehabilitation and Geriatrics, Geneva University Hospitals (HUG), Gabrielle-Perret-Gentil 4, 1205 Geneva, Switzerland; ^2^Department of Radiology, Emergency-Room Radiology Unit, Geneva University Hospitals (HUG), Gabrielle-Perret-Gentil 4, 1205 Geneva, Switzerland; ^3^Department of Internal Medicine, Rehabilitation and Geriatrics, Geneva University Hospitals (HUG) and Geneva Faculty of Medicine, Gabrielle-Perret-Gentil 4, 1211 Geneva, Switzerland

## Abstract

Pulmonary embolism (PE) induces an acute increase in the right ventricle afterload that can lead to right-ventricular dysfunction (RVD) and eventually to circulatory collapse. Hemodynamic status and presence of RVD are important determinants of adverse outcomes in acute PE. Technologic progress allows computed tomography angiography (CTA) to give more information than accurate diagnosis of PE. It may also provide an insight into hemodynamics and right-ventricular function. Proximal localization of emboli, reflux of contrast medium to the hepatic veins, and right-to-left short-axis ventricular diameter ratio seem to be the most relevant CTA predictors of 30-day mortality. These elements require little postprocessing time, an advantage in the emergency room. We herein review the prognostic value of RVD and other CTA mortality predictors for patients with acute PE.

## 1. Introduction

Pulmonary embolism (PE) has a wide spectrum of presentations and severity. Some patients present with shock, requiring urgent thrombolysis [1], while others can be safely treated on an outpatient basis with anticoagulation alone [2]. Guidelines propose tailoring management of PE depending on the risk of adverse outcomes, which depends on hemodynamic status (presence of shock or hypotension), biomarkers (brain natriuretic peptide or cardiac troponin levels), and imagery [1, 3]. Among normotensive patients, right-ventricular dysfunction (RVD) has been shown to carry a higher mortality [4–6]. Echocardiography has become the standard procedure to evaluate RVD but requires skilled specialists and is not available around the clock in many hospitals [7].

Nowadays, computed tomography angiography (CTA) is by far the most commonly used modality to diagnose pulmonary embolism. CTA also allows appreciating vessel and cardiac chamber size. Furthermore, contrast medium flow is a dynamic process. Abnormal flow dynamics can manifest in two ways: diverted hyperdense venous opacification and an altered temporal relationship of vascular opacification [8]. Thus even if CTA produces static images, it provides clues for dynamic or functional parameters, therefore making multislice chest CTA an attractive alternative to echocardiography for prognostic assessment. Using information given by a single test also avoids time consuming and often costly supplemental procedures.

We will herein review indirect hemodynamic signs given by chest CTA and their impact on risk stratification.

## 2. Hemodynamic Consequence of Pulmonary Arterial Obstruction at a Glance

In the pulmonary circulation, cardiac output (3 L/min/m^2^) faces low arterial resistance (80 dynes*·*s/cm^5^) and generates low pressure (mean pulmonary arterial pressure, 15 mmHg). The blood flow can eventually triple to face an increased demand without changing pulmonary pressure by recruitment of new vascular beds.

The burden of pulmonary arterial obstruction and previous cardiorespiratory state determine the hemodynamic consequences of PE. In previously healthy subjects, there is a nonlinear correlation between the degree of pulmonary arterial obstruction and pulmonary pressure. Pulmonary pressure elevation is negligible until obstruction involves more than 30–50% of the arterial bed but increases rapidly above that threshold [9]. This increase is steeper in case of previous pulmonary or left-heart disease. Arterial obstruction and reflex vasoconstriction induced by hypoxia or locally released cytokines cause pulmonary hypertension and enlargement of proximal arterial vessels [10].

**Figure 1:**
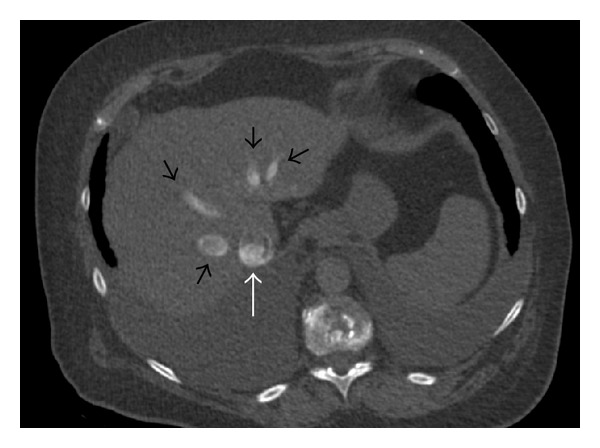
Computed tomography showing significant (grade 5) reflux of contrast media in the inferior vena cava (white arrow) and hepatic veins (black arrows) seen in a 75-year-old man diagnosed with pulmonary embolism and right-ventricular dysfunction.

Increased central venous return related to hypoxia-induced peripheral venoconstriction in addition to reflex tachycardia increases right-ventricular preload and stroke volume. Therefore, in medium-sized PE, cardiac output remains normal or even slightly increased despite the higher afterload. However, when the right ventricle (RV) can no longer accommodate this pressure increase, signs of right-ventricular failure occur. The blood stasis will cause vein enlargement (e.g., superior and inferior vena cava and azygos vein). Contrast medium reflux in the inferior vena cava on chest CTA (Figure 1) is an indirect sign of tricuspid valve insufficiency with elevated right atrial pressure [11].

**Figure 2:**
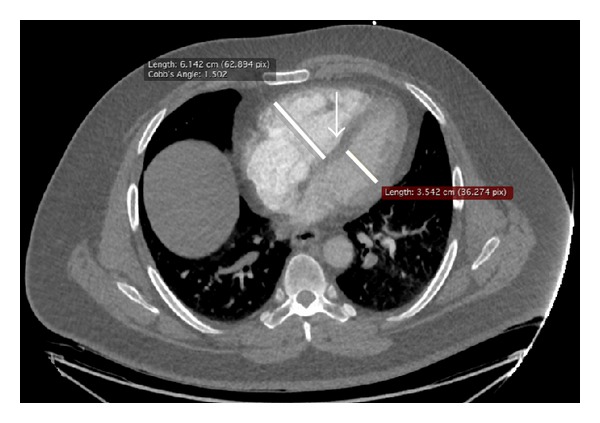
Computed tomography of a 75-year-old man with right-ventricular dysfunction showing increased right-to-left diameter ratio. The short-axis is measured at the widest points between the inner surface of the free wall and the surface of the interventricular septum, in axial transverse images used to diagnose procedure, without reconstructions. The right and left ventricle diameters selected to calculate the ratio are by definition the largest transverse diameters and are therefore often measured in different CTA slices. Note the interventricular septum bowing to the left (arrow).

The right-ventricular wall is thin, in comparison to that of the left ventricle. Wall stress generated by an acute increase in mean pulmonary arterial pressure above 40 mmHg results in right-ventricular dilatation (Figure 2) [9]. RV pressure and dilatation can induce a shift of the septum that will abnormally bow to the left (Figure 2). Because the pericardium is inextensible, this results in an acute decrease of left-ventricular (LV) compliance. In addition, a decrease in coronary blood flow leads to myocardial ischemia that first affects the RV due to an already increased oxygen demand and then exacerbates abnormal LV compliance. The decrease in stroke volume of the RV can lead to a decrease in pulmonary venous return to the left ventricle, causing a drop in systemic blood pressure. Syncope or shock occurs in 5–10% of patients with acute PE [12].

Chest CTA gives indirect signs of right-heart afterload (size of the main pulmonary arteries and emboli burden) or preload (size of azygos vein and vena cava) and allows an estimate of right-ventricular dysfunction through right-to-left ventricular ratios (diameter, surface, volume, or even function), interventricular septum bowing, and retrograde reflux of contrast into the veins. All these signs are interdependent (e.g., right-to-left ventricle ratios and embolic burden or retrograde reflux of contrast into the veins) and may give information on more than one of the following physiologic entities: preload, afterload, and ventricular function.

## 3. Computed Tomography Angiography Signs of Right-Ventricular Dysfunction

In the late nineties, ventricular dilatation [13] and interventricular septum bowing [12] were recognized on helical computed tomography. RV dilatation and LV reduced volume both contribute to a high right-to-left ventricle diameter ratio [14].

### 3.1. Right-to-Left Ventricular Ratios

Right ventricle dilatation can be assessed by different methods on CTA. The right-to-left ventricular diameter ratio is more commonly used because it is simple to measure and mirrors the concept of right ventricle dilatation at echocardiography. The fastest method is to measure the heart chambers' minor axis at the widest points between the inner surface of the free wall and the surface of the interventricular septum in the same images used for diagnosis, without reconstructions (Figure 2). The right and left ventricle diameters selected to calculate the ratio are by definition the largest transverse diameters and are therefore often measured in different CTA slices. However, “submassive pulmonary embolism,” as defined by the American Heart Association criteria, requires the use of reconstructed images in order to obtain a four-chamber view, comparable to echocardiography [3, 15]. The latter method is more time consuming and is dependent on manipulations of workstation by the radiologist that can result in different divergent planes of four-chamber views and its incremental value is limited [16, 17]. The European guidelines do not define the method for calculating the right-to-left ventricle diameter ratio [1].

Since ventricles have a complex tridimensional shape that is not considered when the ratio is measured in two dimensions, some authors have proposed to measure volumetric ratios [18, 19]. This volume ratio might be more accurate in predicting mortality [20]. However, it demands manual outlining of endocardial contour, which needs an additional 4 to 11 minutes [18], a practical issue limiting its generalisation. A further step for improving the assessment of heart function by CTA is to synchronize it with the ECG in order to obtain diastolic and systolic ventricular images. Stroke volume and ejection fraction can be calculated accurately with a good reproducibility compared to MRI [21]. However, ECG-driven CTA did not demonstrate a statistically different area under the receiver-operator curve compared to axial ratio for predicting adverse clinical outcomes [22]. This technique results in an extra amount of contrast agent and radiation exposure. Ejection fraction derived from the ECG-synchronized CTA is time consuming (around 20 minutes [21]) and requires expertise [22].

Indeed, all those methods (axial ratio with or without reconstruction and volumetric ratio) can be used to measure ventricular dilatation and stratify the risk associated with PE, with a good interobserver reproducibility (kappa > 0.8 for RVD and Spearman's rank correlation > 0.8) [16, 17]. In their meta-analysis on the association between right-ventricular dysfunction and mortality, Becattini et al. found similar results for the different methods used to estimate the right-to-left ventricle ratio [17].

Studies exploring CTA include very different patient populations with a mortality ranging from 5 to 18% [4]. Also, the thresholds used to define RVD are not uniform. The most frequently used cut-off point for axial and four-chamber view right-to-left ventricular ratio is 1.0 [4] and 1.2 for volume ratio [18]. Higher cut-off points select a population at increased risk of death [17]. The American Heart Association recommends a cut-off of 0.9 while the ESC proposes a cut-off of 0.9 or 1.0 for the right-to-left ventricle diameter ratio [1, 3]. Whatever the cut-off used is, overall RVD assessed by CTA is observed in more than 50% of patients diagnosed with PE [4]. In their meta-analyses, Becattini et al. [17] and Trujillo-Santos et al. [4] confirmed an increased mortality associated with RVD in all-comers [17] and in the normotensive subset of patients with pulmonary embolism [4, 17]. The risk of death at 30 days after diagnosis doubles (Table 1) for patients with an increased right-to-left ventricle ratio (diameter, surface, or volume ratio) determined by CTA, a result comparable to RV dysfunction assessed by echocardiography [5, 6]. The absolute risk increases from 5.1% (105/2049) to 11.2% (293/2612) in all-comers including patients with shock and from 3.3% (33/984) to 5.4% (69/1270) in normotensive patients with an increased right-to-left ventricle ratio [17].

### 3.2. Septum Bowing

This nonspecific sign of increased right-sided pressure is found roughly in 20% of patients with PE (Figure 2) [14, 16, 27]. Septum bowing has an excellent specificity (100%) but a poor sensitivity (26%) for predicting RV dysfunction [11]. Furthermore, it is the right-ventricular CTA sign with the poorest interobserver reproducibility (kappa: 0.32), thus limiting its clinical application [16]. It has been shown to confer a greater risk of ICU admission [27, 28] and short-term death (Table 1) [17, 18].

## 4. Computed Tomography Angiography Estimate of Afterload

### 4.1. Embolic Obstruction Burden Score and Localization

The number of emboli and their locations are expected to correlate with prognosis. Computed tomography angiography allows an accurate visualisation of emboli up to the subsegmental portions of the pulmonary arteries [29]. Many different CTA scores integrate the number of occluded vessels and the degree of obstruction (complete versus incomplete) with conflicting results concerning their association with death. Vedovati et al. found no association with mortality [23]. However, this recent meta-analysis explored only one scoring system, the Qanadli score [30], and was limited by a small number of studies and a high degree of heterogeneity with many outliers. Moreover, all emboli burden scores are laborious to perform and simpler information can be readily obtained on CTA, namely, the central position (main or lobar arteries) of the emboli. The central location of the emboli seems to have a greater influence on the risk of 30-day mortality than the presence of multiple minor emboli (included in burden scores) [23]. This is confirmed in the same meta-analysis (Table 1) [23].

### 4.2. Pulmonary Artery Size

In small heterogeneous populations, the diameter of the main pulmonary artery [26] and the ratio between the pulmonary artery and the ascending aorta were proposed as indicators of pulmonary hypertension [10], since the size of the main pulmonary artery increases in severe PE [28, 31]. The diameter of the main pulmonary artery and the ratio between the pulmonary artery and the ascending aorta failed to demonstrate a correlation with mortality in the context of acute PE in a large study and meta-analysis [14, 23].

### 4.3. Blood Flow Distribution on Dual-Energy CTA

Standard CTA uses a single X-ray beam at a fixed potential (single-energy), which gives useful structural information, but is sometimes limited in differentiating between tissues with similar attenuation. The principle of dual-energy imaging has been established a long time ago [32] but was only recently implemented on modern CT scanner devices [33–35]. Dual-energy CT analyses simultaneously the X-ray attenuation at low- and high-energy levels (usually 80 and 140 kV), which brings specific information about a particular structure or tissue (e.g., used to differentiate calcium from iodine, iodine from blood clot, and so forth). Dual-energy chest CTA has many applications [35]. In suspected acute PE, it provides excellent morphologic details while allowing the identification of small peripheral thrombi [36].

By isolating the iodine component of the tissue, dual-energy CTA gives the distribution of contrast medium within the lung parenchyma (parallel to the blood perfusion of the lung). Lung perfusion scores derived from dual-energy CTA and number of pulmonary segments with reduced blood flow have been shown to correlate with CTA obstruction score [37–40], troponin I [38], D-dimer [24], and right-to-left ventricular ratio [24, 37–42]. Only one retrospective study suggested a link with mortality (7/18 deaths in the group with a perfusion defect of more than 5% and 5/35 death in the group with smaller defect, Table 1) [24]. Another study showed greater perfusion defects among the 10/60 patients admitted to ICU or dying from PE compared to normotensive patients with PE [37]. However, the dual-energy CTA increases the postprocessing time, the number of images to be stored, and requires expertise. Numerous artefacts also limit its interpretation (e.g., heart and diaphragmatic movement and parenchymal abnormalities) [35]. Irradiation dose seems not to be increased compared to single-energy CTA but depends on type of dual-energy (single source or dual source) and protocol used [35, 43]. The usefulness of dual-energy CTA in risk stratification for acute PE deserves further study.

## 5. Computed Tomography Angiography Estimate of Preload

Preload is more difficult to appreciate on CTA. Saugel et al. found a disappointing correlation between invasive measurement (transpulmonary thermodilution) and CTA determined parameters of left-sided hemodynamics (preload and lung water content) [44]. Contrast reflux in vena cava and vein size integrate information on ventricular pressure overload, dilatation, and decreased function. These are arbitrarily classified as preload for the conceptual purposes of this paper. Cardiac chambers are also related to preload.

### 5.1. Retrograde Reflux of Contrast into the Veins

Reflux of medium contrast into the inferior vena cava (IVC) is an indirect CTA sign of increased RV pressure that can be seen in various underlying conditions (Figure 1) [8]. It is present in around 20% of patients with acute PE [25]. The severity of the reflux of contrast medium can be graded as 1 = no reflux to 6 = reflux into IVC with opacification that extends down to the distal hepatic veins [25]. Only reflux down to the hepatic veins (grade ≥ 4) seems to have prognostic significance [25]. Interobserver reproducibility is good when high grades of reflux are considered (≥4) (Kappa: 0.57–0.78) [16, 25]. Reflux of contrast medium into the IVC is a significant predictor of RVD determined by echocardiography or biomarkers (94% sensitivity and 55% specificity) [11] and of 30-day mortality (Table 1) [18].

### 5.2. Vein Size

An increased pressure in the right atrium results in the widening of dependent veins such as the azygos vein, coronary sinus, and superior and inferior vena cava (IVC) [28, 45]. All of these vein sizes were significantly different in patients with RVD assessed by echocardiography compared to those without [45]. IVC size incorporated in a multivariate model predicted better RVD than as a single parameter or ratio measured on CTA [45]. In a small retrospective study, the azygos vein and SVC sizes were different between deceased patients and survivors from an acute PE [31]. However evidence is lacking on the real utility of those CTA parameters to predict 30-day mortality.

## 6. Clinical Implication for PE Risk Stratification

An ideal risk stratification tool should be able to identify patients at higher risk of death deserving admission to a monitored unit and/or thrombolysis and safely select low-risk patients eligible for short hospital stay or outpatient treatment.

As previously discussed, the most validated CTA sign is the right-to-left ventricle diameter ratio, other signs having no utility (obstruction score), lacking evidence (contrast reflux) or having a high interobserver variability (septum bowing) (Table 1). However the absolute 30-day mortality risk increases only slightly for patients with an increased right-to-left ventricle ratio [17]. Thus, the pooled estimated positive predictive value for right-to-left ventricle diameter ratio measured in transverse images is only 10% (95% CI 6–15%) [17]. With a positive likelihood ratio of 1.3 (95% CI 1.1–1.4) this result has little utility in practice, since the posttest probability is only little different from the pretest probability. CTA performances are comparable to those of echocardiography, which thus adds little for further risk stratification after CTA. Some data suggest that combination of CTA with cardiac biomarkers [20, 46] or clinical scores may further optimize risk stratification [47, 48]. Nevertheless, in the recently published PEITHO study in which the intermediate-risk population was selected based on evidence of both myocardial injury (elevated troponin) and RVD assessed by CTA or US, the 30-day mortality in the heparin-alone arm was low (2.8%) [46].

The pooled estimated negative predictive value for right-to-left ventricle diameter ratio measured in transverse images is 95% (95% CI 93–97%) [17], making CTA a good candidate to identify low-risk patients. However, the negative likelihood ratio is only 0.7 (95% CI 0.6–0.9) [4], a result comparable to echocardiography (NLR 0.6 (95% CI 0.4–0.9)) [5]. This apparent discrepancy between high negative predictive value and poorly discriminative NLR is due to the low overall mortality associated with PE. Therefore, it should be recognized that a number of patients with PE and no RVD on CTA are still at risk of dying. On the other hand, a simple score, the PESI score, based only on clinical variables, is able to accurately identify patients with a 30-day mortality below 3% and has been extensively validated. Therefore, risk stratification by imaging whatever the index used is not clinically relevant for identifying low-risk patients eligible for short hospital stay or outpatient treatment [2].

In summary, available evidence suggests a role for CTA as an alternative to echocardiography for identifying patients with intermediate-risk PE eligible for closer monitoring. In contrast, low-risk patient should be identified with PESI score (I or II) [2].

## 7. Conclusion

This review shows the prognostic values that can be gathered on an already available CTA, which is (fast) always done for diagnosis. However, the choice of CTA or echocardiography for risk stratification remains dependent on their institutional availability. Right-to-left short-axis ventricular ratio is the most relevant CTA-parameter for predicting short-term mortality (within 30 days) and can replace echocardiography for RVD assessment. However, its incremental value for risk stratification in acute PE is low (likelihood ratios close to 1).

Further research should focalize on management strategies depending on CTA-based risk categories, in combination with clinical scores or biomarkers. Although proximal location of emboli, septum bowing, and reflux of contrast medium into the hepatic veins may be of interest, there is insufficient evidence to recommend their clinical use. Right-to-left short-axis ventricular ratio measurement on CTA demands little postprocessing time, a useful feature in the emergency room.

## Figures and Tables

**Table 1:** Computed tomography angiography (CTA) signs, involved mechanism, association with short-term mortality, and level of evidence.

CTA sign	Pathophysiology	Proportion of patients with PE and positive sign	30-day mortality OR (95% CI)	Data based on	Interobserver variability^∗^	Level of evidence^†^
Main pulmonary artery size	Extension of arterial obstruction and pulmonary hypertension/right ventricular afterload	Variable	Not statistically significant	4 small retrospective studies and two meta-analyses [17, 23]	Fair	Low
Emboli burden			Not statistically significant	Meta-analysis of 9 studies [23]	Fair	Good
Emboli position			2.2 (1.3–3.9) for main or lobar arteries localisation	Meta-analysis of 3 studies [23]	Excellent	Good
Blood flow on dual-energy CTA			3.8 (1.0–14.6)^‡^ for a defect >5%	2 small retrospectives studies	Unknown	Low

Right-to-left ventricular ratio	Right-ventricular dysfunction	>50%	2.1 (1.6–2.8) for all-comers with pulmonary embolism	One meta-analysis (>5000 patients) [17]	Excellent	Good
			1.7 (1.1–2.7) for normotensive patients	Two meta-analyses (>2000 patients each) [4, 17]	Excellent	Good
Interventricular septal bowing		20%	1.8 (1.2–2.7)	One meta-analysis (1422 patients) [17]	Poor	Low

Retrograde reflux of contrast	Tricuspid regurgitation, increased atrial pressure/right- ventricular preload	20%	3.1 (1.2–7.7)^§^	>6 small and 1 intermediate-size retrospective study	Fair-excellent	Low
Azygos vein size		Variable	1.5 (1.1–2.0)^||^	1 small retrospective study	Fair	Low

*Based on kappa statistic: <0.4 poor; 0.4–0.75 fair; >0.75 excellent; ^†^global appreciation of scientific evidence based on the number, size, quality of the studies, and availability of a meta-analysis; ^‡^calculated from Bauer et al. [24]; ^§^calculated from Aviram et al. [25]; ^||^14-day mortality [26].

CTA: computed tomography angiography; OR: odds ratio; 95% CI: 95% confidence interval.
